# Dietary patterns and risk of colorectal cancer in Tehran Province: a case–control study

**DOI:** 10.1186/1471-2458-13-222

**Published:** 2013-03-12

**Authors:** Akram Safari, Zalilah Mohd Shariff, Mirnalini Kandiah, Bahram Rashidkhani, Foroozandeh Fereidooni

**Affiliations:** 1 Department of Nutrition and Dietetics, Faculty of Medicine and Health Sciences, Universiti Putra Malaysia, Selangor, Malaysia; 2 Department of Community Nutrition, School of Nutrition and Food Science, Shaheed Beheshti University of Medical Sciences, PO Box 19826–19573, Tehran, Iran; 3 Department of Surgical Oncology, Cancer Institute, Imam Khomeini Hospital Complex, Tehran University of Medical Sciences, Tehran, Iran

**Keywords:** Western dietary pattern, Healthy dietary pattern, Colorectal cancer, Case–control study, Iranian adults

## Abstract

**Background:**

Colorectal cancer is the third and fourth leading cause of cancer incidence and mortality among men and women, respectively in Iran. However, the role of dietary factors that could contribute to this high cancer incidence remains unclear. The aim of this study was to determine major dietary patterns and its relationship with colorectal cancer.

**Methods:**

This case–control study was conducted in four hospitals in Tehran city of Iran. A total of 71 patients (35 men and 36 women, aged 40–75 years) with incident clinically confirmed colorectal cancer (CRC) and 142 controls (70 men and 72 women, aged 40–75 years) admitted to hospital for acute, non-neoplastic diseases were recruited and interviewed. Dietary data were assessed by 125-item semi-quantitative food frequency questionnaire. Multivariate logistic regression was used to estimate the relationship between dietary patterns and risk of colorectal cancer.

**Results:**

Two major dietary patterns (Healthy pattern and Western pattern) were derived using principal component analysis. Each dietary pattern explained 11.9% (Healthy pattern) and 10.3% (Western pattern) of the variation in food intake, respectively. After adjusting for confounding factors, the Healthy dietary pattern was significantly associated with a decreased risk of colorectal cancer (OR= 0.227; 95% CI=0.108–0.478) while an increased risk of colorectal cancer was observed with the Western dietary pattern (OR=2.616; 95% CI= 1.361-5.030).

**Conclusion:**

Specific dietary patterns, which include healthy and western patterns, may be associated with the risk of colorectal cancer. This diet-disease relationship can be used for developing interventions that aim to promote healthy eating for the prevention of chronic disease, particularly colorectal cancer in the Iranian population.

## Background

In 2008, colorectal cancer (CRC) was estimated to be the third and second most commonly diagnosed cancer in the world with 10.0% and 9.4% of total cancer cases among males and females, respectively [1,2]. Although the rates of CRC in Africa and Asia are lower as compared to CRC rates reported in Europe, North America, New Zealand and Australia [3], the former tend to increase with migration and westernization. In Asia, higher rates of CRC are observed in Japan and China [4]. Among Iranians, CRC is the third and fourth most commonly diagnosed malignancy in males and females, respectively. In the last few decades, the incidence and mortality rates of CRC have increased markedly in Iran [5,6].

Family history, unfavourable socioeconomic conditions, tobacco smoking, alcohol consumption, obesity, physical inactivity, cooking techniques and various aspects of diet have been associated with the risk of CRC [7,8]. A number of studies have assessed the relationship between diet and risk of CRC, but they mainly focused on the intake of single food items or nutrients [9,10]. Although such analyses are valuable, they give inadequate information on diet and disease relationship. As people consume meals that consist of a complex combination of variety of nutrients and non-nutrients that are interactive or synergistic with each other, it is difficult to isolate the effect of a single nutrient or food on health [11]. Examination of patterns of dietary intake (multiple dietary components) rather than focusing on the individual nutrients is recommended as a complementary and suitable approach for clarifying relationships between diet and health [11,12]. Most of the existing data on cancers and dietary patterns were collected from developed countries, with almost two-thirds of these surveys conducted in either North America or Europe [13]. There is a lack of published data on this topic in Iran as a developing country. The aim of this study was to identify dietary patterns and its association with the risk of CRC in Tehran, Iran.

## Methods

### Subjects

This hospital-based case–control study was conducted from September 2008 through January 2010 in 19 CRC surgical units of the Cancer Institute of Imam Khomeini Hospital Complex and three major general hospitals (Shariati, Imam Hussein and Ayatollah Taleghani) in Tehran city, Iran. Cases were patients with pathologically confirmed CRC, diagnosed no longer than six months before the interview, aged 40–75 years of age at the time of diagnosis and had no previous diagnosis of cancer at other sites, prior history of inflammatory bowel disease or familial adenomatous polyposis. Controls were selected randomly from patients admitted to the same hospitals as cases during the same time period for acute, non-neoplastic conditions and not afflicted with diet related chronic diseases. They were admitted to the hospitals for various medical conditions (38% for fractures and sprains, 14.1% for osteoarticular disorders, 11.3% for disk disorders, 9.8% for acute surgical conditions e.g., hernia inguinalis, appendicitis and kidney stone, 7.0% for trauma and injuries, 7.0% for skin diseases and 12.0% for other illnesses e.g., eye or nose disorders, debridement, removal of plates, pins, screws and wires). Each patient with CRC was matched with two patients in the control group by age (within 5-year categories) and sex.

Of 267 patients (89 cases and 178 controls) screened for the study based on inclusion and exclusion criteria, 16 controls and 8 cases were excluded. During dietary pattern analysis, 30 more patients (10 cases and 20 controls) with incomplete food frequency questionnaire (> 40% of food items were not assessed) and total energy intakes were outside the range of ± 3 standard deviation from the mean (< 716 and > 3,764 Kcal/d for men and <541 and >3,397 Kcal/d for women) were excluded. The final sample for statistical analysis was 71 cases and 142 controls. On average, less than 20% of cases and controls were ineligible to participate to the study.

### Dietary assessment

Dietary intake was assessed with a semi-quantitative food frequency questionnaire that consisted of 125 foods and beverages (with standard serving sizes) commonly consumed by Iranians. Diets of respondents were based on intakes during the year prior to cancer diagnosis (cases) or interview (controls). This FFQ has been shown in previous studies to have a good validity and reproducibility [14,15]. A validated food album [16] and a set of household measurements (e.g., cup, tablespoon, teaspoon plate, glass, small bowl, and spatula) were used to assist respondents to estimate the portion size and type of food items. Respondents were asked to report the frequency of consumption of a given serving of each food item on a daily, weekly, monthly and yearly basis and data were then converted to daily intake frequency. Portion size of each food item consumed was converted to grams. Intake of each food item in grams was then determined by multiplying the portion size by daily intake frequency. Consumption of seasonal food items (e.g., watermelon, melon, and persimmon) was taken into account based on the period of the year the food items were available. The edible fraction of foods was also considered using household measurement guidelines [16]. Total energy intake was estimated by adding the energy value of each food in the FFQ. Food energy value was based on the Nutrients Composition of Iranian Foods [17] and the USDA Food Composition Data [18]. The latter was used for foods or food ingredients that were not available in Nutrients Composition of Iranian Foods.

To identify dietary patterns, the 125 food items were categorized into 31 food groups (Table 1) based on their similarity of nutrient content and culinary usage or their relationship with cancer [13]. Food items that were not fit to be included in a certain food group or were assumed to represent individual dietary behaviours were left as unique food groups (e.g. fried potato, mayonnaise, egg, tea and doogh or Iranian yogurt drink) [13].

**Table 1:** Food groups used in the dietary pattern analysis

**Food group**	**Food items**
Processed meat	Sausages, hamburger, salami
Red Meat	Beef, mutton, ground meat ,Visceral meat
Fish	Tuna, any type of fish
Poultry	Chicken
Egg	Fried eggs, boiled eggs
Low fat dairies	Low fat milk, Low fat yogurt, ordinary yogurt,
High fat dairies	Whole milk, yogurt( high fat, drained and cream), cream cheese, cream, ice cream
Yogurt drink	Yogurt drink
Tea	Black tea, green tea
Coffee	Coffee
Fruits	Cantaloupe, watermelon, melon, sloe, apple, apricot, cherry, sour cherry, fig, nectarine, peach, pear, Citrus fruit, date, kiwi, grape, pomegranate, strawberry, banana, grape fruit, plum, persimmon, raisin, mulberry, compotes, other fruits
Artificial juice	Lemon juice and packed juice
Tomato	Tomato
Carrot	Carrot
vegetables	Spinach, lettuce, mixed vegetable, stew vegetables, eggplant, green squash, local vegetables, pepper, mushroom, cucumber, garlic, kinds of cabbage, root vegetables, other vegetables
Legumes	Bean, chickpea, split pea, soybean, lentil, other cereals
Fried potato	Fried potato
Boiled potato	Boiled potato
Whole grains	Barbari bread, Sangak bread, Taftoon bread, local bread
Refined grains	Lavash bread, baguette, rice, macaroni
Snacks	Biscuits, puff, chips
Nuts	Peanut, almond, walnut, pistachio, hazelnut, roasted seeds
Sweets and desert	Cakes, cookies, chocolate, pastry, dry sweet, honey, jam, halvah
Sugar	Sugar, sugar cube, candy, sugar candy, tahini
Pickles	pickle, cucumber Pickle
Animal butter	Animal butter
Solid oil	Solid vegetable oil, animal fat, rump
Liquid oil	liquid oil
Olive	Olive and olive oil
Mayonnaise	Mayonnaise
Soft drink	Carbonated drinks

### Covariates

Cases and controls were interviewed by trained interviewers using pre-tested questionnaires. Information obtained include socio-demographic characteristics, family history of CRC, physical activity, smoking habit, medication information (use of non-steroidal anti inflammatory drugs (NSAIDs), vitamin/mineral supplements), cooking techniques and dietary intake. Alcohol intake information was not sought from respondents as during the pre-testing of questionnaire, they refused to respond to the item due to cultural and religious beliefs. Iran is an Islamic country where sale and consumption of alcoholic beverages are prohibited.

Weight was measured to the nearest to 0.1 kg with respondents wearing minimal clothes and without shoes. For patients who had undergone surgery or a long stay in the hospital, the weight at the time of admission was used as current weight. Height was measured using a SECA body metre with a precision of 0.1cm with respondents stand erect, without shoes, their feet together and eyes in a parallax state. For bedridden patients, the recumbent length was obtained in a supine position. Body mass index (BMI) was then calculated as weight (in kilograms) divided by height (in meters) squared and was classified according to the World Health Organization’s standard for adults [19]. A validated self-report questionnaire was used to measure physical activity level of respondents in metabolic equivalent task (MET) hours/day, on the basis of reported time spent on different activities which were weighted according to intensity level [20,21]. Physical activity was completed based on activities during the year before CRC diagnosis (cases) or during the year before interview (controls).

### Statistical analysis

Data were analyzed with Statistical Package Software for Social Science, version 16 (SPSS Inc., Chicago, IL, USA). Kolomogorov-Smirnov test was used to check for data normality. Square root data transformation was applied for data not normally distributed.

Dietary patterns were derived using Principal Component Analysis (PCA) based on the 125 food items. Prior to extracting factors (patterns), suitability of using factor analysis for this study was assessed using two criteria tests, namely Kaiser-Meyer-Olkin (KMO-test) and Bartlett’s test of sphericity. Sampling adequacy and inter-correlation of variables were supported by KMO value > 0.647 and Bartlett’s test of sphericity < 0.05, respectively. Communality index was assessed to indicate the variance in each food group being explained by the analysis [22,23]. Food groups (e.g. chicken, egg, boiled potato and high fat dairy) with communality value < 6% indicated low and insufficient degree of correlation with other food groups and were thus dropped from subsequent analysis. Scree plot was assessed to determine the number of factors with an eigenvalue of > 1.0, and Varimax rotation was applied to review the correlations between variables and factors [22,23]. Post-rotated factor loadings showed two dietary patterns described the sample and these patterns were labelled based on each food group having the highest loading on each pattern. Food groups with positive loadings in each pattern indicate the direct relationship with that pattern and food groups with negative loadings shows the inverse relationship with that pattern. The factor score for each pattern was calculated by summing the consumption of each food group that were weighted by factor loading and each person received an individual factor score for each identified pattern [24]. Factor scores were then categorized into two groups based on the mean of factor score (0) and used as the outcome variable.

Chi-square (χ2) test was used to assess the associations between variables and study groups. Independent t-test was applied for comparing means between two groups. Uni-variate and multivariate logistic regression were used to estimate the odds ratio with a 95% confidence interval for risk factors of CRC. A p-value <0.05 that is based on a two-sided statistical test was considered as significant.

### Ethical considerations

The present study was approved by the Medical Research and Ethics Committee of Universiti Putra Malaysia, Faculty of Medicine and Health Sciences and the Ministry of Health, Treatment Medical and Education of Iran. Written informed consents were obtained from all respondent prior to the interviews.

## Results

Table 2 shows the socio -demographic and lifestyle characteristics of the71 cases and 142 controls. By frequency matched design, age and gender were similar in controls and cases. The mean age of respondents at diagnosis of colorectal cancer (CRC) was 59.9 years for men and 55.6 years for women.There were no statistically significant differences between case and control groups by educational level, occupation, income, physical activity (MET. h/day), cooking methodsor smoking status. However, cases were more likely than controls to have a family history of CRC in the first (p=0.004) and second degree relatives (p=0.003). Of the four main types of non-steroidal anti-inflammatory drugs (NSAIDs) which were commonly used by respondents, only aspirin (p= 0.012) and acetaminophen (p= 0.049) were significantly different between case and control groups. Compared to the cases, controls were more likely to take mineral supplements (25.3% versus 11.3%; p=0.017). The mean body mass index (BMI) was slightly higher in the case than in the control group, but it did not differ statistically. Cases had significantly higher energy intake than the controls (p=0.045).

**Table 2:** Characteristics of case and control groups

**Variable**	**Control (N=142) n (%)**	**Case (N=71) n (%)**	**P-value
Gender			
Female	72(50.7)	36(50.3)	*
Male	70(49.3)	35(49.3)	
Age (years)			
Male (M ± SD)	59.93±10.46^**†**^	59.9 ± 10.14^**††**^	*
Female (M ± SD)	55.99±10.29	55.65 ±10.36	
Level of education			0.147
No formal education	36(25.4)	28 (39.4)	
Elementary	45(31.7)	22 (31.0)	
Junior/Senior high school	19 (9.2)	7 (9.9)	
Diploma/ College/University	42(29.6)	14 (19.7)	
Occupation			0.776
Employed/Government	14( 9.9 )	6 (8.5)	
Employed/Private	15(10.6)	10(14.1)	
Housewife(unemployed )	58(40.8)	33(46.5)	
Retired	26(18.3)	11(15.5)	
Self-employed	29(20.4)	11(15.5)	
Total household Income			
< $ 425	83(58.5)	46(78.8)	
≥ $ 425	59(41.5)	25(21.2)	
M ± SD	471.07 ± 285.4	413.20 ± 237.7	0.141
Family history of CRC in first degree	2(1.4)	7 (9.9)	**0.004**
Family history of CRC in second degree	1(0.7)	6 (8.5)	**0.003**
Smoking status			0.948
Never	103(72.7)	50 (70.6)	
Former smoker, (pack/year) < 20	9 (6.3)	7 (9.8)	
Former smoker, (pack/year) ≥ 20	8 (5.6)	2 (2.8)	
Current smoker, (pack/year) < 20	13 (9.1)	5 (7.0)	
Current smoker, (pack/year) ≥ 20	9 (6.3)	7 (9.8)	
Physical activity			0.498
Active	73 (51.4)	33 (46.5)	
Sedentary	69 (48.6)	38 (53.5)	
Common ways of cooking meat			0.282
Fried	28 (19.7)	20 (28.2)	
Fried /Boiling	71 (50.0)	35 (49.3)	
Smoking/Grilling	43 (30.3)	16 (22.5)	
Common ways of preparing vegetable			0.148
Raw/ fresh	78 (54.9)	29 (40.8)	
Boiled	18 (12.7)	8 (11.3)	
Fried, Fried / freezed	46(32.4)	34(47.9)	
Non-steroidal anti-inflammatory drugs use			
Ibuprofen	22 (15.5)	5 (7.1)	0.081
Aspirin	16(11.3)	1(1.4)	**0.012**
Acetaminophen	29(20.42)	4(5.6)	**0.049**
Baby aspirin	19(13.4)	15(21.1)	0.146
Vitamin use	18(12.7)	7(9.85)	0.547
Mineral use	36(25.3)	8(11.3)	**0.017**
Body mass index (Kg/m2)			
≤ 24.9	48(33.7)	22(32.8)	
> 24.9	94(66.3)	49(67.2)	
Mean BMI	26.67± 4.2	27.23 ± 4.2	0.361
Energy Intake (kcal/d) (M ± SD)	2063 ± 40.7	2212 ± 50.7	**0.045**

*Matched variables of the study ^**1**^USD= 9,980.00 Rial Significant difference at p< 0.05 M ± SD: Mean ± Standard deviation.

^**1**^MET: metabolic equivalent task (1MET=energy expenditure of sitting quietly or approximately 1kcal/kg of body weight per hour).

**P-values were estimated using chi-square (χ^2^) statistics, independent t-test for the difference between case and control groups.

†Meanage at the interview.

†† Mean age at the diagnosis.

Factor analysis revealed two dietary patterns and the factor loadings for each dietary pattern are presented in Table 3. Food groups with absolute factor loadings > 0.20 were considered as having significant contribution to the pattern. These two dietary patterns explained 22.20% of the total variance in food intake. The first pattern with high loadings for fruits, vegetables, liquid oil, olive, fish, yoghurt drink, whole grains, carrot, low-fat dairy products and nuts was labelled “ Healthy “ dietary pattern. This pattern was significantly related to respondents who were non-married, had diploma or higher degree, self-employed, with higher income, use acetaminophen, vitamins and commonly consume raw/ fresh vege. The second pattern which loaded heavily on sugar, processed and red meat, animal butter, refined cereals, tea, pickles, solid oil, mayonnaise, soft drink, legumes, sweets and desserts, and snacks was named “Western” dietary pattern. This pattern was related to being married, current smoker and not using vitamins and aspirin and with high energy intake.

**Table 3:** Factor loading matrix of food groups for Healthy and Western dietary patterns

**Food group**	**Healthy pattern**	**Western pattern**
Vegetables	0.707	
Fruits	0.682	
Olives	0.582	
liquid oils	0.561	
All Fish	0.526	
Low fat Dairy	0.479	
Carrot	0.477	
Nuts	0.441	
Whole Grains	0.381	
Yogurt Drink	0.294	
Sugars		0.585
Red meat		0.540
Pickles		0.496
Refined Grains		0.495
Soft Drink	−0.276	0.490
Animal butter		0.479
Mayonnaise		0.458
Black tea		0.430
Processed Meat		0.408
Legumes		0.347
Solid Oil		0.328
Sweets and desert		0.278
Snacks		0.270
Total variance	11.92%	10.28%

Absolute factor loading values < 0.20 for both patterns were excluded for simplicity.

The odds ratio and their 95% confidence interval for CRC by the mean of dietary pattern scores are presented in Table 4. After adjusting for family history of CRC in first and second-degree relative, energy intake, cooking of vegetables, aspirin, acetaminophen and mineral supplement use, the Healthy dietary pattern was associated with a decreased risk of colorectal cancer. Those having “high” healthy dietary pattern (high consumption of healthy foods) had lower risk of CRC (OR= 0.227, 95% CI=0.108–0.478). An increased risk of colorectal cancer was associated with “high” Western dietary pattern in that those consuming more western foods had higher risk of CRC (OR=2.616; 95% CI =1.361–5.030).

**Table 4:** Odds ratios and 95% confidence intervals for colorectal cancer by dietary patterns

**Dietary pattern**	**Contrls n(%)**	**Cases n(%)**	**Crude OR**	**95% CI**	*Adjusted OR	**95%CI**
**Healthy dietary pattern**						
† Low	65(45.8)	52(73.2)	1.00		1.00	
†† High	77(54.2)	19 (26.8)	0.308	0.166-0.574	0.227	0.108-0.478
P-value			**<0.001**		**<0.001**	
**Western dietary pattern**						
Low	87(61.3)	28(39.4)	1.00		1.00	
High	55(38.7)	43(60.6)	2.429	1.355-4.354	2.616	1.361-5.030
P-value			**0.003**		**0.004**	

*Adjusted for family history of CRC in first and second-degree relative, vegetable preparation, aspirin, acetaminophen, mineral and energy intake.

† Respondents with score < 0 (mean factor score = 0) on a dietary pattern.

†† Respondents with score > 0 on a dietary pattern.

## Discussion

In this case–control study of Iranian adults in Tehran, Iran, a Western dietary pattern (WDP) was associated with a greater risk of CRC while a Healthy dietary pattern (HDP) conferred a protective effect against CRC risk after adjustment of socio-demographic and lifestyle factors. The findings suggest that the associations between these dietary patterns and risk of CRC are independent of other risk factors of CRC.

Although consuming certain food items such as solid oil, sweets and sugar that are representative of WDP might explain the relationship with CRC, its mechanism remains unknown. The most likely mechanism is through overweight and obesity, which are important risk factors for CRC [25]. In a cross-sectional study in Iran,the adoption of a Western pattern, which was characterized by a higher intake of butter, high-fat dairy products, sweets and desserts, hydrogenated fats, soft drinks, potatoes, pizza, red and processed meat was positively related to obesity [26]. Murtaughand co-workers [27] found the animal protein pattern was associated with a more than threefold increased risk of obesity, while the prudent or healthy dietary pattern showed a 29% decreased risk of obesity among Hispanic women.

The CRC and dietary pattern relationship observed in our study was comparable to those identified in previous studies. In a study among the US population [12], the Prudent pattern, characterized by a high consumption of vegetables, fruits, whole grains, poultry, fish, and legumes, was inversely associated with the risk of CRC while the Western pattern (high intake of sweets and desserts, red and processed meats, refined grains and french fries) was positively associated with this malignancy. Other studies have also reported that a dietary pattern characterized by low intake of fried foods, alcohol and processed meat or high intake of fruits, vegetables, yams, cereals, legumes and low fat dairy products lowered the risk of CRC [28-30].

In the present study, fruits, vegetables and whole grains were related to HDP. These food groups have been shown to have a protective effect against CRC through various mechanisms [31,32]. They are good sources of various micronutrients and other compounds including minerals, vitamins A, C, E, carotenoids, selenium, flavonoids, and fibres, which display overlapping and complementary mechanisms of action, such as binding and dilution of carcinogens and anti-oxidant effects [31,33]. Higher intake of dietary fibre and whole grains may bind and dilute potential carcinogens in stool and speed up their transit through the intestines [32,33]. These actions may consequently change the colonic flora to an environment that is protective against CRC.

It has been hypothesized that high fat intake, through its consequent high caloric intake and obesity, may potentially cause hormonal changes that can over motivate the re-generation of colonic endothelial cells and persuade the growth of polyps and adenomas [34,35]. It may also promote CRC risk through its altered immunologic responses and insulin resistance [12,34]. Several studies have reported that it is the source of fat as well as the type of fat that is associated with the occurrence of CRC [36,37]. For example, the protective role of olives against CRC could be due to the type of polyunsaturated fatty acids (PUFAs) and the existence of some micro-nutrients like vitamin E, poly phenols and various anti-oxidant components [12,38,39]. In this study, consumption of olive oil and liquid oil was related to HDP while the consumption of solid oil and animal butter was related to WDP.

A number of studies [10,40] showed that the intake of milk and milk products is negatively associated with CRC risk. The protective effect of milk and dairy products can be attributed to the existence of some micronutrients such as riboflavin, calcium, vitamin B12and vitamin D. This food group also contains other protective substances such as butyric acid, linoleic acid, sphingomyelin and probiotic that can reduce cancer risk [41]. The relationship between dairy products and CRC may also be influenced by the fat content. While high fat dairy products increased the risk of CRC [39,42], low fat dairy products lowered the risk of CRC [43,44]. Similar to other studies [39,42], the present study found that high intake of low-fat dairy products contributed to HDP.

Fish consumption has been recognized in dietary patterns as a protective factor for CRC [12,39]. This inverse association could be attributed to the high intake of n-3 polyunsaturated fatty acids and vitamins D and A from fish. In the present study, fish was a component in HDP. The traditional Iranian diet does not entail high consumption of fish due to the high prices and/or seasonal availability [45]. However, Tehran has a significantly larger fish consuming population compared to other provinces of Iran [46].

In some studies, tea consumption is a risk factor for CRC [47,48] while in others [12,39], an inverse association was observed between tea intake and CRC risk. The inconsistent finding of tea consumption might be related to constipation and the inverse association with secretion of bile acids [49]. In the present study, tea had a high factor loading for Western (unhealthy) dietary pattern. In Iran, black tea is the most popular drink that is commonly served with sugar or cube sugar. This study also showed that sugar group (e.g. sugar, sugar cube, candy, sugar candy, tahini) had the highest factor loading for WDP as compared to other food groups in this pattern.

Similar to other studies [38,42] our study suggested that a high consumption of red or processed meat elevates the risk of CRC. A meta-analysis of 19 cohort studies also revealed a positive association between high intake of red meat with CRC risk [50], This effect can be attributed to the meat content of saturated fatty acids and heme iron as well as the formation of N-nitroso-compounds (NOCs), polycyclic aromatic hydrocarbons (PAHs), heterocyclic amines (HCAs) and acryl amide as a result of higher temperatures during cooking [7,51]. The relationship between meat and cancer risk could depend on the cooking or preservation method of meats [51].

Surprisingly, we also found that intake of legumes was observed in WDP [37]. The findings on legumes and CRC risk are mixed with Fung et al., [12] reported that high intake of legumes in the prudent pattern decreased the risk of CRC while Kesse and colleagues [38] failed to show any significant relationship between legumes and CRC risk. At present, we do not understand the positive association between legumes and CRC risk but it may be due to the preparation method of legume dishes that could diminish the favorable effect of legumes. In Iran, important legumes such as lentil, chickpea, pea and different varieties of bean are commonly prepared with rice, meat and vegetables [52]. A popular traditional lentil soup served for breakfast is usually prepared with animal butter and fried onion. Red bean and chickpea are used in *ghorme sabzie* and *ghymeh khoresht,* respectively*.* In *ghorme sabzie*, fried vegetable (using solid oil) and red meat are common ingredients while in *ghymeh khoresht,* fried chickpea is mixed with red meat, fried onion and potatoes [52]. It should be noted that in this study, solid oil, animal butter and red meat groups had higher factor loadings in WDP.

This study has several limitations. First, the possibility of selection bias cannot be avoided in retrospective case–control studies. The present study minimized this problem by matching the cases and controls by age and sex. In addition, both groups were distributed similarly by hospital status [29]. There is also a possibility that individuals with colorectal cancer would recall their diets differently than controls as a result of their disease status [53]. As cases were selected not more than 6 months of cancer diagnosis and during the interview, cases were reminded to report foods consumed prior to diagnosis, these could minimize the possibility of cases reporting post-diagnosis. Also, our findings that Western dietary pattern was associated with increased risk of CRC and Healthy dietary pattern reduced the risk of CRC could further corroborate the non-difference in diet recall between cases and controls. Second, this study could not assess the long term effects of risk factors on the incidence of colorectal cancer. A cohort or longitudinal study is a better alternate for the development of chronic diseases such cancer and can determine the associations with socioeconomic, lifestyle behaviours and anthropometry. Third, the different stages of factor analysis utilized in this study included the grouping of food items, determining the number of factors, Eigenvalue, method of rotation and labelling of factors. These interpretations were subjective as they were based on the decision of the researcher guided by previous published methods of analysing dietary patterns. Finally, the sample size of the current study was relatively small and the study was only conducted on people living in Tehran city. These could limit the generalization of study findings to the entire Iranian population. Despite these limitations, this is the first study in Tehran province in the northeast of Iran to examine the relationship of major dietary patterns and colorectal cancer risk among middle age adults.

## Conclusion

The present study suggested that a diet characterized by high consumption of fruits, vegetables, liquid oil, olive, carrot, fish, yoghurt drink, whole grains, low-fat dairy products and nuts (labelled as healthy diet) was associated with lower risk of colorectal cancer, while a diet with high intake of sugar, processed and red meat, animal butter, refined cereals, tea, pickles, solid oil, mayonnaise, soft drink, legumes, sweets and desserts and snacks might increase the risk of colorectal cancer. The study also supported the importance of using dietary pattern method to investigate the compound relationship between diet and colorectal cancer. This diet-disease relationship can be used for developing interventions that aim to promote healthy eating for the prevention of chronic diseases, particularly colorectal cancer in the Iranian population.

## Competing interests

All authors declare that they have no conflict of interest.

## Authors’ contributions

AS, contributed to the conception and design of the study, collected the data, analyzed and interpreted data and drafted the manuscript. ZMS, contributed to the conception and design of the study, data analysis and interpretation, critically reviewed the manuscript and gave final approval to publish the manuscript. M K, contributed to the conception and design of the study. B R, participated in study design, data analysis and interpretation. FF, assisted in data collection. All authors read and approved the final manuscript.

## Pre-publication history

The pre-publication history for this paper can be accessed here:

http://www.biomedcentral.com/1471-2458/13/222/prepub
